# Supplementary material for: Virus and cell specific HMGB1 secretion and subepithelial infiltrate formation in adenovirus keratitis
Source: PLoS Pathog. 2025 May 14;21(5):e1013184. doi: 10.1371/journal.ppat.1013184 (PMC12101768; doi:10.1371/journal.ppat.1013184)
Supplement: S1 Table — (DOCX) [file ppat.1013184.s008.docx]

**Minimal data set**

Data for Figure 1B

| Cell type | Subcellular location | Time (hrs) | Mock | | HAdV-D37 | |
| --- | --- | --- | --- | --- | --- | --- |
|  |  |  | Mean | SD | Avg | Mean |
| THE | Nuclear | 2 | 5412776 | 2315919 | 8071709 | 3548170 |
|  |  | 12 | 4279496 | 915860 | 7259422 | 3219685 |
|  |  | 24 | 5234033 | 1876566 | 1152953 | 512952 |
|  |  | 48 | 11325701 | 3709307 | 314213 | 226619 |
|  | Cytoplasmic | 2 | 448568 | 403449 | 2676670 | 1229469 |
|  |  | 12 | 543342 | 483860 | 4899645 | 2979148 |
|  |  | 24 | 808231 | 396930 | 12063934 | 6289721 |
|  |  | 48 | 1158929 | 534786 | 10022852 | 877688 |
| PCEC | Nuclear | 2 | 10332485 | 7971572 | 2813337 | 1971437 |
|  |  | 12 | 14227421 | 10571526 | 3531526 | 2525039 |
|  |  | 24 | 14139875 | 10047358 | 3122496 | 2022373 |
|  |  | 48 | 14734583 | 10419413 | 5225583 | 3795770 |
|  | Cytoplasmic | 2 | 13863099 | 3717421 | 1974327 | 640320 |
|  |  | 12 | 14928092 | 2754696 | 2450117 | 417730 |
|  |  | 24 | 23498109 | 17618764 | 2669839 | 2905258 |
|  |  | 48 | 2838267 | 663065 | 12181271 | 224046 |
| HCF | Nuclear | 2 | 35602808 | 11491581 | 34765886 | 9898510 |
|  |  | 12 | 33521290 | 10258404 | 32247529 | 8926344 |
|  |  | 24 | 43056480 | 11242165 | 18655957 | 11539412 |
|  |  | 48 | 42669488 | 9192227 | 26592860 | 23408980 |
|  | Cytoplasmic | 2 | 10788641 | 3979615 | 12841907 | 4526132 |
|  |  | 12 | 10335897 | 3871280 | 8736875 | 8151534 |
|  |  | 24 | 19902075 | 5601545 | 15005521 | 5340440 |
|  |  | 48 | 7866277 | 2270020 | 14280228 | 9820496 |
| A549 | Nuclear | 2 | 26720657 | 306285 | 26700720 | 2218839 |
|  |  | 12 | 31566388 | 12727689 | 34927996 | 5261425 |
|  |  | 24 | 28620521 | 9431099 | 26882716 | 1849456 |
|  |  | 48 | 31279545 | 11644646 | 37251596 | 13343962 |
|  | Cytoplasmic | 2 | 34048257 | 9752991 | 28939069 | 11107178 |
|  |  | 12 | 38449381 | 11197315 | 45275754 | 8863244 |
|  |  | 24 | 40617538 | 20814566 | 39827389 | 28958239 |
|  |  | 48 | 28949245 | 13842952 | 75363170 | 18449308 |
| HEK293 | Nuclear | 2 | 30557764 | 5313907 | 28182994 | 8863956 |
|  |  | 12 | 28303467 | 6231652 | 12038985 | 7893056 |
|  |  | 24 | 25504960 | 4922206 | 12053507 | 6857080 |
|  |  | 48 | 22444867 | 4179172 | 10843587 | 9525037 |
|  | Cytoplasmic | 2 | 23091839 | 4153105 | 31808636 | 5040256 |
|  |  | 12 | 24389559 | 2646566 | 16269779 | 2316182 |
|  |  | 24 | 17746373 | 1986663 | 20481262 | 1437959 |
|  |  | 48 | 19742631 | 3581927 | 18140484 | 1923371 |
| Statistical method used | | p values were determined by t test; *p < 0.05, **p < 0.01, ***p < 0.001, ****p < 0.0001 (n=3) | | | | |

Data for Figure 1C

| Cell type | Time (hrs) | Mock | | HAdV-D37 | |
| --- | --- | --- | --- | --- | --- |
|  |  | Mean | SD | Mean | SD |
| THE | 2 | 102098.6667 | 111783.4044 | 198200.7 | 56160.66 |
|  | 12 | 137828.3333 | 92371.16066 | 303503.7 | 107818 |
|  | 24 | 315151 | 290648.7875 | 1193227 | 298557 |
|  | 48 | 427810.3333 | 258534.9184 | 5557092 | 1289776 |
| HCF | 2 | 69778.66667 | 91276.5093 | 756427.7 | 338120.7 |
|  | 12 | 81106 | 65080.99356 | 1048591 | 393972.8 |
|  | 24 | 97026.66667 | 58644.84508 | 1398914 | 413180.7 |
|  | 48 | 101362.3333 | 61795.16216 | 9258915 | 3222066 |
| Statistical method used | | p values were determined by t test; *p < 0.05, **p < 0.01, ***p < 0.001, ****p < 0.0001 (n=3) | | | |

Data for Figure 2B

|  | Mean | SD |
| --- | --- | --- |
| Mock | 0.135789 | 0.099546 |
| 6 hrs | 0.092596 | 0.088879 |
| 8 hrs | 0.607266 | 0.239843 |
| 10 hrs | 0.85258 | 0.141599 |
| 12 hrs | 0.938288 | 0.074878 |
| 24 hrs | 0.180577 | 0.118112 |
| Statistical method used | p values were calculated by one-way ANOVA (Tukey's multiple comparisons test) | |

Data for Figure 2C

| Time (hrs) |  | Mean | SD |
| --- | --- | --- | --- |
| 6 | Mock | 0.135789 | 0.099546 |
|  | HAdV-D37 | 0.092596 | 0.088879 |
| 8 | Mock | 0.135789 | 0.099546 |
|  | HAdV-D37 | 0.607266 | 0.239843 |
| 10 | Mock | 0.135789 | 0.099546 |
|  | HAdV-D37 | 0.85258 | 0.141599 |
| 12 | Mock | 0.133362 | 0.099160 |
|  | HAdV-D37 | 0.938288 | 0.074878 |
| 24 | Mock | 0.829080583 | 0.130181 |
|  | HAdV-D37 | 0.180577 | 0.118112 |
| Statistical method used | Statistical testing performed using unpaired t-test (two-tailed).  Analysis was done on 60,000 cells/group (n=5) | | |

Data for Figure 4D

|  | Mean | SD |
| --- | --- | --- |
| Mock | 0.293393 | 0.1644 |
| HAdV-D37 | 0.805922 | 0.1569 |

Data for Figure 5D

|  | Mean | SD |
| --- | --- | --- |
| 3 hpi | 0.093 | 0.058 |
| 6 hpi | 0.028 | 0.040 |
| 9 hpi | 0.112 | 0.056 |
| 12 hpi | 0.363 | 0.046 |
| 24 hpi | 2.785 | 0.454 |
| 36 hpi | 7.285 | 3.000 |
| 48 hpi | 10.186 | 0.217 |

Data for Figure 6B

| 12 hrs | | Nuclear | | Cytoplasm | |
| --- | --- | --- | --- | --- | --- |
|  |  | Mean | SD | Mean | SD |
| NC-siRNA | Mock | 0.280508333 | 0.066852552 | 0.216895 | 0.092676731 |
|  | HAdV-D37 | 0.372599 | 0.328164648 | 0.829908667 | 0.147552039 |
| siCRM1 | Mock | 0.881921 | 0.120402648 | 0.090182667 | 0.029126639 |
|  | HAdV-D37 | 0.797457667 | 0.104879733 | 0.028538667 | 0.025243423 |
| Statistical method used | | Analyzed by ANOVA with Tukey’s post-hoc test | | | |

Data for Figure 6D

| 12 hrs | | Nuclear | | Cytoplasm | |
| --- | --- | --- | --- | --- | --- |
|  |  | Mean | SD | Mean | SD |
| NC-siRNA | Mock | 0.755054 | 0.028493325 | 0.317935 | 0.08686684 |
|  | HAdV-D37 | 0.303092333 | 0.073006062 | 0.659074667 | 0.03312719 |
| siLAMP1 | Mock | 0.574843333 | 0.059249685 | 0.178411 | 0.027196268 |
|  | HAdV-D37 | 0.245729667 | 0.060865449 | 0.544367667 | 0.078792934 |
| Statistical method used | | Analyzed by ANOVA with Tukey’s post-hoc test | | | |

Data for Figure 6F

| 24 hrs | | Nuclear | | Cytoplasm | |
| --- | --- | --- | --- | --- | --- |
|  |  | Mean | SD | Mean | SD |
| NC-siRNA | Mock | 0.766582 | 0.194401535 | 0.6960315 | 0.010312473 |
|  | HAdV-D37 | 0.18773275 | 0.11642083 | 0.209593 | 0.080182687 |
| siLAMP1 | Mock | 0.70857975 | 0.104194876 | 0.68238575 | 0.104292658 |
|  | HAdV-D37 | 0.55985425 | 0.179295672 | 0.526293 | 0.130413796 |
| Statistical method used | | Analyzed by ANOVA with Tukey’s post-hoc test | | | |

Data for Figure 7B

|  | Buffer | | rHMGB1 | |
| --- | --- | --- | --- | --- |
|  | Mean | SD | Mean | SD |
| CXCL5 | 29.134 | 3.016753 | 36.278 | 1.381687 |
| CXCL1 | 26.9 | 5.690324 | 44.2535 | 6.538616 |
| HGF | 4.924 | 2.121320344 | 17.222 | 6.363961031 |
| IGFBP-2 | 61.803 | 1.414213562 | 109.928 | 0.212132034 |
| IGFBP-3 | 62.878 | 1.984141628 | 66.4825 | 5.891613701 |
| IL-6 | 15.553 | 4.242641 | 64.973 | 11.66726 |
| IL-8 | 157.978 | 2.12132 | 189.178 | 0.707107 |
| MCP-1 | 52.658 | 5.656854 | 136.9805 | 2.828427 |
| MCP-3 | 26.563 | 1.414214 | 121.358 | 1.414214 |
| MIF | 0.7225 | 1.414214 | 28.9585 | 0.707107 |
| Osteopontin | 43.233 | 4.949747 | 67.158 | 7.071068 |
| Pentraxin 3 | 21.048 | 2.12132 | 70.893 | 1.414214 |
| SDF-1a (CXCL12) | 4.508 | 1.414214 | 21.191 | 7.424621 |
| Thrombospondin-1 | 77.788 | 5.656854 | 126.728 | 3.535534 |
| VEGF | 100.643 | 3.53553 | 162.323 | 0.00 |

Data for Figure 7D

|  | Control 1 | Control 2 | Control 3 | rHMGB1 1 | rHMGB1 2 | rHMGB1 3 |
| --- | --- | --- | --- | --- | --- | --- |
| Dkk-1 | -0.977 | -0.960 | -0.711 | 0.206 | 1.766 | 0.676 |
| ENA-78 | -0.653 | -1.261 | -1.009 | 0.756 | 0.932 | 1.235 |
| ENDOGLIN | -0.480 | 1.186 | 0.656 | -1.919 | 0.497 | 0.059 |
| GROa | -1.690 | -0.246 | -0.668 | 0.438 | 1.307 | 0.859 |
| HGF | -1.095 | -0.858 | -0.884 | 0.361 | 1.448 | 1.028 |
| IGFBP-2 | -1.123 | -0.731 | -0.996 | 1.443 | 0.435 | 0.970 |
| IGFBP-3 | -0.668 | -0.008 | -0.039 | 1.855 | -1.424 | 0.284 |
| IL-6 | -1.113 | -0.880 | -0.982 | 0.785 | 1.154 | 1.036 |
| IL-8 | -1.281 | -0.775 | -0.909 | 0.956 | 0.988 | 1.020 |
| MCP-1 | -1.088 | -0.852 | -1.052 | 0.983 | 1.012 | 0.996 |
| MCP-3 | -1.048 | -0.957 | -0.993 | 1.012 | 0.977 | 1.009 |
| MIF | -0.970 | -0.951 | -1.071 | 1.106 | 0.890 | 0.995 |
| Osteopontin | -0.638 | -1.443 | -0.816 | 1.101 | 0.793 | 1.003 |
| Pentraxin 3 | -0.938 | -1.029 | -1.032 | 1.000 | 0.999 | 1.000 |
| SDF-1a | -1.215 | -0.608 | -1.052 | 0.547 | 1.301 | 1.027 |
| Thrombospondin-1 | -0.906 | -1.119 | -0.966 | 1.078 | 0.910 | 1.002 |
| u PAR | -1.054 | -0.978 | -0.962 | 0.890 | 1.027 | 1.077 |
| VEGF | -1.296 | -0.673 | -0.979 | 0.884 | 1.049 | 1.014 |
| ANGIOGENIN | 0.753 | 1.130 | 1.093 | -0.948 | -1.074 | -0.954 |
| ANGIOPOIETIN-1 | 0.873 | 1.129 | 0.988 | -1.048 | -1.009 | -0.933 |
| ANGIOPOIETIN-2 | 0.986 | 0.873 | 1.100 | -1.137 | -1.137 | -0.684 |
| BDNF | 1.712 | 0.019 | 0.873 | -0.758 | -1.042 | -0.804 |
| CHITINASE 3-LIKE 1 | 0.382 | 1.471 | 0.990 | -0.861 | -0.946 | -1.036 |
| Serpin E1 | 1.041 | 0.908 | 1.022 | -0.808 | -1.252 | -0.911 |
| Statistical method used | Heatmap rows depict z-score SD variation from the mean value for each cytokine (n=3) | | | | | |

Data for S1 Figure A

| Time (hrs) |  | Mean | SD |
| --- | --- | --- | --- |
| 2 | Mock | 0.575191 | 0.055514 |
|  | HAdV-D37 | 0.465288 | 0.103204 |
| 12 | Mock | 0.716943 | 0.045737 |
|  | HAdV-D37 | 0.637217 | 0.395327 |
| 24 | Mock | 0.485041 | 0.016948 |
|  | HAdV-D37 | 0.675647 | 0.200194 |
| 48 | Mock | 0.368208 | 0.130964 |
|  | HAdV-D37 | 0.415757 | 0.153595 |

Data for S1 Figure B

| Time (hrs) |  | Mean | SD |
| --- | --- | --- | --- |
| 2 | Mock | 0.629871 | 0.098108 |
|  | HAdV-D37 | 123515.3 | 123187.3 |
| 12 | Mock | 0.949602 | 0.790144 |
|  | HAdV-D37 | 234785 | 212331.6 |
| 24 | Mock | 0.862335 | 0.684008 |
|  | HAdV-D37 | 415147.4 | 587104.8 |
| 48 | Mock | 0.764456 | 0.419711 |
|  | HAdV-D37 | 473004.8 | 122280.9 |

Data for S2 Figure A

| Time (hrs) |  | Mean | SD |
| --- | --- | --- | --- |
| 6 | Mock | 0.14161 | 0.100149 |
|  | Infection | 0.09259 | 0.081135 |
| 8 | Mock | 0.14161 | 0.100149 |
|  | Infection | 0.60726 | 0.218946 |
| 10 | Mock | 0.14161 | 0.100149 |
|  | Infection | 0.85258 | 0.129262 |
| 12 | Mock | 0.18786 | 0.096146 |
|  | Infection | 0.93828 | 0.071690 |
| 24 | Mock | 0.07886 | 0.053315 |
|  | Infection | 0.18057 | 0.113083 |

Data for S2 Figure B

| Time (hrs) |  | Mean | SD |
| --- | --- | --- | --- |
| 0 | Nucleus | 0.830184 | 0.245687 |
|  | Cytoplasm | 0.140217 | 0.137364 |
| 6 | Nucleus | 0.508322 | 0.19405 |
|  | Cytoplasm | 0.252264 | 0.083869 |
| 8 | Nucleus | 0.45749 | 0.257589 |
|  | Cytoplasm | 0.471014 | 0.051377 |
| 10 | Nucleus | 0.321413 | 0.252285 |
|  | Cytoplasm | 0.673913 | 0.101297 |
| 12 | Nucleus | 0.22028 | 0.195013 |
|  | Cytoplasm | 0.950634 | 0.041547 |
| 24 | Nucleus | 0.107843 | 0.140817 |
|  | Cytoplasm | 0.585809 | 0.355577 |

Data for S3 Figure B

|  | Mock | | HAdV-D37 | |
| --- | --- | --- | --- | --- |
|  | Mean | SD | Mean | SD |
| EMMPRIN | 35.8865 | 1.634124 | 91.4715 | 0.451841 |
| IL-1α | 3.181 | 0.156978 | 32.0585 | 0.596091 |
| IL-1ra | 2.099 | 0.076368 | 14.6095 | 0.1492 |
| FGF-19 | 3.1235 | 1.240972 | 6.9465 | 0.359917 |
| IL-8 | 1.87 | 0.082024 | 64.5765 | 0.90439 |
| IP-10 | 1.2085 | 0.055861 | 142.572 | 1.658873 |
| I-TAC | 1.3335 | 1.096723 | 81.7575 | 5.65049 |
| MIF | 27.4965 | 1.438962 | 77.5485 | 3.145918 |
| MIP-3α | 0.147 | 0.123037 | 12.143 | 1.042275 |
| RANTES | 2.7705 | 0.666802 | 26.0005 | 1.707663 |
| VEGF | 16.2745 | 4.95894 | 59.2755 | 8.49306 |

Data for S4 Figure B

|  | Mock | | HAdV-D37 | |
| --- | --- | --- | --- | --- |
|  | Mean | SD | Mean | SD |
| CYSTATIN C | 17.7645 | 0.876105 | 34.419 | 1.582505 |
| Dkk-1 | 8.719 | 0.944695 | 62.542 | 3.251277 |
| EMMPRIN | 42.393 | 1.005506 | 72.618 | 0.612354 |
| CXCL5 | 4.761 | 0.072125 | 35.463 | 0.002828 |
| FGF-7 | 17.4875 | 4.872673 | 49.81 | 1.957272 |
| FGF-19 | 3.0955 | 0.006364 | 11.436 | 0.165463 |
| IL-1α | 2.861 | 0.234759 | 24.0665 | 0.907218 |
| IL-8 | 87.7775 | 1.276328 | 123.4295 | 0.597505 |
| IP-10 | 2.757 | 0.531744 | 91.2825 | 2.359615 |
| MIF | 35.5135 | 1.349867 | 55.057 | 1.525936 |
| MIP-3α | 11.92 | 2.660136 | 15.0305 | 2.615588 |

Data for S5 Figure E

|  | Mean | SD |
| --- | --- | --- |
| Mock | 3.435 | 0.8131 |
| HAdV-D37 | 10.76 | 0.5656 |
