# Supplementary material for: Virus and cell specific HMGB1 secretion and subepithelial infiltrate formation in adenovirus keratitis
Source: PLoS Pathog. 2025 May 14;21(5):e1013184. doi: 10.1371/journal.ppat.1013184 (PMC12101768; doi:10.1371/journal.ppat.1013184)
Supplement: Attachment — Submitted filename: Saha HMGB1 Response to Reviewers_.docx [file ppat.1013184.s010.docx]

**Point-by-point responses**

**Prior Manuscript Submission #:** PPATHOGENS-D-25-00122
**Title:** Virus and Cell Specific HMGB1 Secretion and Subepithelial Infiltrate Formation in Adenovirus Keratitis
PLOS Pathogens

**From the Editor:**

Thank you for submitting your manuscript to PLOS Pathogens. After careful consideration, we feel that it has merit but does not fully meet PLOS Pathogens's publication criteria as it currently stands. Therefore, we invite you to submit a revised version of the manuscript that addresses the points raised during the review process.

*RESPONSE: Thank you for the opportunity to resubmit our manuscript. We hope it exceeds expectations.*

* A rebuttal letter that responds to each point raised by the editor and reviewer(s). You should upload this letter as a separate file labeled 'Response to Reviewers'. This file does not need to include responses to any formatting updates and technical items listed in the 'Journal Requirements' section below.
* A marked-up copy of your manuscript that highlights changes made to the original version. You should upload this as a separate file labeled 'Revised Manuscript with Track Changes'.
* An unmarked version of your revised paper without tracked changes. You should upload this as a separate file labeled 'Manuscript'.

*RESPONSE: These have been uploaded as requested.*

**Additional Editor Comments:** The reviewers requested additional chromatin fractionation experiments to show the localization of HMGB1 (cytoplasm, nucleus) and a timecourse for the 3D infections. In addition, please be sure to provide the requested loading controls for experiments in the manuscript as well as a better and more representative image for Figure 3F. Please be sure to address all of the comments made by all three reviewers in the revised manuscript.

*RESPONSE: To address the time point concern, we have now added an additional supplementary figure (S6), to show 3D infections at a later time post infection (24 hpi). This supplements Figure 8 from the original submission, which shows images from 12 hpi. The 3D culture system is delicate, time-consuming, and expensive, and we relied on extensive time course experiments in cell culture to determine the optimal time points for 3D culture. We find these two time points (12 and 24 hpi) relevant based on our results from Fig 2, where we show maximum HMGB1 release at 12 hpi and 24 hpi. In 2D cell culture experiments, there was no HMGB1 release at earlier times pi. We placed the 12 hpi construct images in the main manuscript because the stroma remained intact at that time and the migrating cells were very visible, mimicking the natural disease with multiple foci of immune cells. The structural integrity of the constructs at 24 hpi was diminished likely due to cytopathic effect and to the direct effects of immune cell migration and their expression products. If the editors believe that we should also show earlier time points, we will comply. If so, we will need additional time to conduct the experiments.*

*With regard to chromatin fractionation, it is unclear but the third reviewer appears to be asking for experiments focused on the potential interaction(s) between protein VII and HMGB1 in the cell nucleus. Our existing data shows convincingly that protein VII does not sequester HMGB1 in the cell nuclei of HAdV-D37 infected human corneal epithelial cells, or else we could not have shown translocation to the cytoplasm or extracellular secretion prior to cytopathic effect. Understanding mechanistically the differences in protein VII interaction with HMGB1, comparing HAdV-C5 and D37 infections, is a project unto itself, one of high interest and which we intend to pursue. However, a thorough study of this issue seems to us to be beyond the scope of the currently submitted manuscript.*

*Every other concern and critique is addressed fully, as outlined below. We have repeated Fig. 3F, and resolved all other figure-related concerns. Fig 3F, Fig 4C, Fig 5B, C, and Fig 8C were all revised. A new Fig S6 was added. All the bioinformatic figures were improved for resolution.*

**Journal Requirements:**

1) We ask that a manuscript source file is provided at Revision.

*RESPONSE: Done*

2) Please provide an Author Summary.

*RESPONSE: Done*

3) We do not publish any copyright or trademark symbols that usually accompany proprietary names, eg ©,  ®, or TM  (e.g. next to drug or reagent names).

*RESPONSE: We have removed all trademarks.*

4) Please upload all main figures as separate Figure files in .tif or .eps format. For more information about how to convert and format your figure files please see our guidelines: 
<https://journals.plos.org/plospathogens/s/figures>

*RESPONSE: We have uploaded all the figures in .tif format.*

5) We notice that your supplementary Figures are included in the manuscript file. Please remove them and upload them with the file type 'Supporting Information'. Please ensure that each Supporting Information file has a legend listed in the manuscript after the references list.

*RESPONSE: We have removed the supplementary Figures from the manuscript file and uploaded them as individual files in .tif format.*

6) Some material included in your submission may be copyrighted. According to PLOSu2019s copyright policy, authors who use figures or other material (e.g., graphics, clipart, maps) from another author or copyright holder must demonstrate or obtain permission to publish this material under the Creative Commons Attribution 4.0 International (CC BY 4.0) License used by PLOS journals. Please closely review the details of PLOSu2019s copyright requirements here: PLOS Licenses and Copyright. If you need to request permissions from a copyright holder, you may use PLOS's Copyright Content Permission form.
Please respond directly to this email and provide any known details concerning your material's license terms and permissions required for reuse, even if you have not yet obtained copyright permissions or are unsure of your material's copyright compatibility. Once you have responded and addressed all other outstanding technical requirements, you may resubmit your manuscript within Editorial Manager. 
Potential Copyright Issues:

- Please confirm that you are the photographer of Figures 8A and 9., or provide written permission from the photographer to publish the photos under our CC BY 4.0 license.

- Figure 9. Please confirm whether you drew the images / clip-art within the figure panels by hand. If you did not draw the images, please provide a link to the source of the images or icons and their license / terms of use; or written permission from the copyright holder to publish the images or icons under our CC BY 4.0 license.

*RESPONSE:*

- *Fig 8A is an original photograph taken by us in our laboratory.*
- *Thank you for asking about Fig. 9. We neglected to note in the Legend that this was previously published by us (**PMID: 31769017), and adapted to this manuscript as permitted by the journal where initially published..*

*All the computer-drawn figures and sub-figures in the manuscript were generated by us using BioRender.com, with a BioRender Academic Publication License. Biorender as the software is mentioned in the respective figures legends.*

7) We note that your Data Availability Statement is currently as follows: "All data are available without restriction". Please confirm at this time whether or not your submission contains all raw data required to replicate the results of your study. Authors must share the “minimal data set” for their submission. PLOS defines the minimal data set to consist of the data required to replicate all study findings reported in the article, as well as related metadata and methods (<https://journals.plos.org/plosone/s/data-availability#loc-minimal-data-set-definition>).
For example, authors should submit the following data: 
- The values behind the means, standard deviations and other measures reported;
- The values used to build graphs;
- The points extracted from images for analysis..
Authors do not need to submit their entire data set if only a portion of the data was used in the reported study.
If your submission does not contain these data, please either upload them as Supporting Information files or deposit them to a stable, public repository and provide us with the relevant URLs, DOIs, or accession numbers. For a list of recommended repositories, please see <https://journals.plos.org/plosone/s/recommended-repositories>. 
If there are ethical or legal restrictions on sharing a de-identified data set, please explain them in detail (e.g., data contain potentially sensitive information, data are owned by a third-party organization, etc.) and who has imposed them (e.g., an ethics committee). Please also provide contact information for a data access committee, ethics committee, or other institutional body to which data requests may be sent. If data are owned by a third party, please indicate how others may request data access.

*RESPONSE: Data required to replicate the results are included. We have created and now uploaded a Minimal Data Set in tabular form for all figures in which quantitative data was utilized (Table S1).*

8) Please ensure that the funders and grant numbers match between the Financial Disclosure field and the Funding Information tab in your submission form. Note that the funders must be provided in the same order in both places as well.
- State the initials, alongside each funding source, of each author to receive each grant. For example: "This work was supported by the National Institutes of Health (####### to AM; ###### to CJ) and the National Science Foundation (###### to AM)."
- State what role the funders took in the study. If the funders had no role in your study, please state: "The funders had no role in study design, data collection and analysis, decision to publish, or preparation of the manuscript.".
If you did not receive any funding for this study, please simply state: u201cThe authors received no specific funding for this work.u201d

*RESPONSE: This is now corrected in the resubmission***.**

**Reviewers' Comments:**

**Part I - Summary**

**Reviewer #1:** Saha et. al., present the results of a study focusing on mechanisms involved in the formation of corneal infiltrates due to Adenovirus group D viruses. These infiltrates interfere with vision resulting in substantial costs. They hypothesize that infection of corneal epithelial cells results in the release of pro-inflammatory mediators that then act on stromal keratocytes to promote inflammation. This could explain the formation of the infiltrates in the absence of stromal cell infection. They focus on HMGB1 based on previous work. They used various continuous cell lines as well as primary corneal epithelial cells and primary stromal keratocytes and a novel 3D corneal cell culture system. The data indicate cell and virus specific effects on HMGB1 expression and release from cells and show that an Adenovirus C (not associated with ocular infection) behaves very differently in terms of affecting HMGB1. Cytokine profiles are compared and showed a number of pro-inflammatory mediators are induced by HGB1 treatment in stromal cells. In the 3D system they demonstrate that HMGB1 treatment results in chemotaxis of neutrophils into the corneal stroma. Overall the work is comprehensive, well designed, and the data support the conclusions. There are a couple of minor issues that should be considered.

*RESPONSE: We thank the reviewer for the acknowledgment and comments.*

1. The opening sentence of the introduction could be deleted since is it not directly relevant. Just start with the Adenovirus introductory material.

*RESPONSE: We have edited the opening sentence in the Introduction (lines 60-62) for relevance and added two references. Please note that line numbers refer to the Track Changed version uploaded, and for reasons we could not correct are different in the uploaded Clean version of the revised manuscript.*

2. In Figure 8 panel C the IF photos of the infiltration are not very convincing and not quantitative. They provide quantitation using blotting for meyeloperoxidase, however. It might be more convincing to include additional panels or insets at higher magnification showing the infiltrates into the stroma.

*RESPONSE: Thank you, we have included insets with higher magnification to better enable visualization of the infiltrating cells.*

**Reviewer #2:** The is a generally well-written manuscript describing mechanistic studies on a common, virulent and highly contagious type of corneal infection. Specifically, the authors describe a novel molecular mechanism that leads to corneal subepithelial infiltration (SEI) formation, a key event in this infection. The research findings add valuable new insights into the pathogenesis of adenovirus-induced keratitis, and present a new understanding of the immunological processes involved with potentially broader implications. The authors provide substantial evidence that secretion of biologically active HMGB1 by infected corneal cells is specific both to the viral agent and the infected cell type. They show that HAdV-D37 infection of immortalized as well as primary corneal epithelial cells are associated with HMGB1 acetylation, CRM1- dependent nuclear-to-cytoplasmic translocation, and LAMP1-mediated release into cell supernatants, in contrast to infection of the same cells by HADV-C5. An important strength of this study is using the 3-dimensional corneal facsimile to simulate and model natural infection of corneal tissue in addition to a monolayer model.

Although broadly well-designed and supported, several aspects of the manuscript could be improved for clarity and accuracy.

*RESPONSE: We thank the reviewer for their overall assessment and insightful comments.*

**Reviewer #3:** In this study, Saha et al. have examined how Human Adenovirus D regulates HMGB1 to control epidemic keratoconjunctivitis (EKC). They show that HAdV-D induces cell-specific secretion of HMGB1 which is regulated by CRM1 and LAMP1-mediated cellular signals. They then establish a new 3D culture system that mimics corneal architecture, exhibiting HMGB1 release that induce leukocytic infiltrates. These findings uncover new insights into the biology of HAdV, establish new physiologic platforms to mechanistically investigate HAdV biology and develop inhibitors to HAdV infection. The authors present their findings in a logical and easy-to-follow manner, with schematics summarizing the findings of each figure, making the manuscript easy to follow. While the findings of this study are potentially exciting, they will benefit from some orthogonal validation assays and characterization of their new infection models, which are described in the Comments.

*RESPONSE: We thank the reviewer for the assessment and for their helpful comments and suggestions.*

**Part II – Major Issues: Key Experiments Required for Acceptance**

**Reviewer #1**: None.

**Reviewer #2:** None noted

**Reviewer #3:** Fig. 5 – It is unclear why GAPDH is not observed in these samples. Regardless, these western blots require a loading control. The LPS-treated samples should be run with AdV infected THE cells to show the dynamic range of IL1b and IL18 induction. More concerning, however, is that in Fig. 5C the antibody does not seem to detect significant induction of IL1b and IL18 activation by LPS. This discrepancy should be resolved.

*RESPONSE: To clarify for the reviewer, these immunoblots are of supernatants from infected cells, not from cell lysates. There should be only secreted proteins in these samples, and no intracellular proteins except in the setting of cell death. We saw no GAPDH release other than very minimal release by PCEC. To satisfy the concern regarding a loading control, we have added a blot showing a Ponceau S stain for these samples as an extra panel at the bottom of Fig 5B. This blot clearly demonstrates equal loading, the presence of protein, and the absence of GAPDH.*

*Our sample volume for supernatants is high, and cannot be accommodated in a 15 well gel cast, but we have also redone the blot in Fig. 5C. In addition, we wish to point the reviewer to the supplementary data (Figs S3 and S4), in which we show by a second broader methodology (human cytokine array) that these two cytokines are not released by infection.*

Chromatin fractionation studies are needed to determine whether nuclear HMGB1 is sequestered on host chromatin in the presence of protein VII from HAdV-D37 or whether it is remaining in a soluble state in the nucleoplasm. Along those lines, HAdV-D37 protein VII-HMGB1 coIP’s would also be beneficial for demonstrating direct interactions of HAdV-D protein VII with host HMGB1.

*RESPONSE: We agree with the reviewer that chromatin fractionation to further study what happens to protein VII in HAdV-D37 infected cells is of interest. However, it should be clear from our data that upon infection of corneal epithelial cells by HAdV-D37, HMGB1 is secreted and not sequestered. This is despite very convincing data for HMGB1 sequestration with HAdV-C5 infection. We respectfully maintain that the work requested by the reviewer falls beyond the scope of our current project. The protein VII experiments we included in our submitted paper were performed to highlight that HAdV-C5 and D37 appear to have unique biologies, likely also dependent on the infected cell type.*

The findings of the siCRM1/siLAMP studies should be orthogonally validated by fractionation experiments combined with immunoblots to determine whether the HMGB and viral protein VII is associated with host chromatin, in the nucleoplasm or cytoplasm.

*RESPONSE: The focus on protein VII is understandable and we share the interest. We are working on a follow-up study to determine molecular interactions between protein VII and HMGB1 in species D Vs C adenoviruses. The data we show in the current manuscript is highly suggestive of differences. However, this was not the principal focus of the submitted work, which already includes extensive data and analysis.*

A timecourse of the infection of the 3D culture is needed to provide a proper characterization of this exciting new system of viral infection.

*RESPONSE: Thank you for the comment. We performed extensive time-course analyses for HMGB1 release using 2D cell cultures (Figure 2), and we timed the 3D culture experiments based on these data. We now show leukocyte infiltration in the 3D model at 2 time points: 12 hpi (previously submitted Figure 8) and 24 hpi (new Supplementary Figure S6).*

Fig. 3F – the western blot should be rerun to resolve protein VI and VII more clearly.

*RESPONSE: Protein VI and protein VII are ~19kDa and ~22 kDa respectively. We repeated this experiment to better resolve these bands. Please see our new Figure 3F.*

**Part III – Minor Issues: Editorial and Data Presentation Modifications**

**Reviewer #1:** See the comments above.

**Reviewer #2:** General:

The underlying rationale for the choice of MOI used in various experiments, or the potential impact if any of using different levels, is not well explained. For example, in figures 3A and 4B, cell viability and LDH assay, confocal microscopy (line 631) and pyroptosis assay (line 685) used HAdV-D37 infection of cells at an MOI of 1, whereas high content microscopy (line 652) and siRNA lockdown (line 671) used MOI5. Why the choices and do those differences matter?

*RESPONSE: Use of higher MOIs with HAdV-D37 leads to very rapid CPE, more rapidly than with other adenoviruses we have studied, including other HAdV-Ds. All time course studies extending to 48 hrs were carried out at MOI of 1 to permit analysis over time. We performed high content microscopy experiments at MOI of 5, because in pilot experiments, MOI of 5 permitted better visualization of HMGB1. We have added a comment to the Methods to address this question: “with this MOI chosen for these experiments to best enable visualization of HMGB1 secretion over time” (lines 606-607).*

A strength of the paper is the multiplicity of orthogonal approaches taken to discover and define the unique interactions that occur in their infection model, but a weakness is in the logical flow in which these are presented. In some cases, discussion of the results and comparison to other studies would better be reserved for the Discussion section. Specifically, the presentation from the first results “HMGB1 is released by adenovirus infected cells in cell-specific fashion” to the “HMGB1 acetylation is cell and virus specific” flows fairly smoothly and the logic is clear. However, the transition into “HMGB1 secretion upon viral infection is independent of pyroptosis” would benefit from some explanation as to why this is the next logical question to flow from those findings since other mechanisms may also impact HMGB1 secretion.

*RESPONSE: We wish to thank this reviewer for recognition of our application of “orthogonal” approaches to our work. We have revised the section on pyroptosis to address this comment, and we hope better clarify the transition to discuss pyroptosis. Please see lines 252 -254 in particular, but the section as revised is as follows:* “Cell death invariably results in some release of cellular contents into the extracellular milieu, regardless of the death pathway [63-67]. Whether release of HMGB1 specific to HAdV-D37 infection of corneal epithelial cells is a bystander effect of cell death, or a specific cellular pathway unrelated to cell death is unclear. In apoptotic cell death, hypoacetylated HMGB1 remains tightly bound to chromatin and is functionally mute [65]. HMGB1 release through pyroptosis is potently proinflammatory.”

**Specific:**

Lines 114-119: These are not part of the results but could be of greater value in the introduction to explain lines 101 -102 about why potential crosstalk between tissue-specific epithelial cells and fibroblasts is a key issue.

*RESPONSE: We appreciate the observation of the reviewer, and agree with the sense of discontinuity. We have moved the text to the Introduction (new Lines 118-123), with some revision to best integrate into the existing text.*

Lines 163-164: Why were THE rather than PCEP cells chosen to elucidate the timeline of HMGB1 secretion upon HAdV-D37 infection here?

*RESPONSE: Primary corneal epithelial cells are difficult to obtain and work with, and passage poorly. Some lots are not viable when purchased. We did not consider it cost-effective for optimization studies, and used them only as confirmatory and for validation.*

Line 205: Fig 3b should be Fig 3B for consistency

*RESPONSE: Corrected, thank You.*

Lines 206-207: “HMGB1 cytoplasmic translocation occurred with infection by HAdV-D9 (Fig 3B)”. Figure 3B shows that this is also true for HAdV-D37 and HAdV-D56?

*RESPONSE: We believe the reviewer’s concern is addressed with one word addition (“also”) to the sentence. (line 209)*

Lines 212-214/Figure 3D: Did the authors use a reference sequence to make the phylogenetic tree or an outgroup?

*RESPONSE: Outgroup, thank you.*

Lines 218-219/Figure 3E: “The protein VII amino acid identity and similarity scores comparing HAdV-D (types 9, 37, and 56) and HAdV-C (types 2 and 5) ranged between 72.41 to 73.63% and 79.31 to 80.60%, respectively (Fig 3E)” but only the comparison between D37 and C5 is shown in Figure 3E. Potentially, description of the %identity in the text may be enough without the figure.

*RESPONSE: We believe the specific amino acid differences between these two viruses, each representative of disparate adenovirus species, may be informative to some readers.*

Lines 274-284: This section could be clearer and more concise. Since Figure 5A shows a schematic representation of corneal epithelial cell release of HMGB1 based largely on what is known from the literature, highlighting many of the mechanisms that influence HMGB1 secretion, this would seem to be an ideal opportunity to explain why the authors then focused on pyroptosis.

*RESPONSE: This comment is similar to that made above about this section of the Results. We hope that our edits to the language, as detailed above, clarify why we have focused on pyroptosis at this particular point in the paper.*

Lines 327-328: Are the authors implying a distinction between CRM1 and exportin 1 XPO? If not, it would be better to stick to one nomenclature throughout.

*RESPONSE: Corrected for clarity, thank you – the term XPO1 now appears only once (line 99).*

Lines 353/Figure 6: Although the authors mentioned that “HMGB1 was previously shown to partially colocalize in LAMP1 containing vesicles”, there are other pathways and molecules involved not tested that should be considered in the Discussion.

*RESPONSE: We have added to the Discussion to address the reviewer’s suggestion, thank you (lines 474-477).*

Lines 361-362/Figure 6G: As mentioned above, there are other pathways and molecules involved that are not tested. Figure 6G seems to imply that only LAMP1 is involved, which should be clarified.

*RESPONSE: Thank you, this is addressed in the comment just above.*

Lines 375-377/Figure E-H: Authors perform substantial bioinformatic analysis, however the main outcomes of that analysis could be better explained. Further it detracts that the text in these figures is blurred.

*RESPONSE: Thank you, we have added considerably to the Results (Lines 319-358) and Discussion (lines 489-494) to clarify these results and what they may mean.*

*Regarding the image resolution, it appeared poor because for the initial submission, high resolution images were not uploaded. Please see the revised manuscript – if there are any additional concerns with the Figures, we will correct them.*

Line 477, references 80-83: Are there more recent studies that bear on this point?

*RESPONSE: Thank you, these were the most recent and also most rigorous studies in the literature.*

Line 583: Typo, should be C02

*RESPONSE: Corrected, thank you.*

Lines 592-593: streptomycin 100g/ml is likely missing a mu.

*RESPONSE: Corrected, thank you****.***

**Figures:**

Figure 3G: I did not see a reference to this in the text. The figure may not be essential.

*RESPONSE: Thank you for your observation. We have added a reference to the Figure in the text (line 226) and in the Legend for the model’s depiction.*

Figure 4C: Scale and control are missing on the WB.

*RESPONSE: Thank you. This blot was created from concentrated supernatants, not from cell lysates. We have added a panel from the same blot to show nonspecific (ns) protein bands for equal loading.*

Figure 4E: What new results does this figure provide?

*RESPONSE: Respectfully, we have included schematics to highlight and clarify main points developed in each section. In this figure, the schematic is provided to highlight that secretion of HMGB1 was specific to corneal epithelial cells and not the two other epithelial cell types tested.*

Figure 7C (as well as E, G and H): text is difficult to read.

*RESPONSE: Thank you. Please let us know if with uploading of the high resolution images fails to solve the resolution problem.*

**Supplemental Figures:**

S1C: Please add scale and positive control in the WB.

*RESPONSE: These blots are same as seen in Fig 1A (with β-actin and TBP loading controls shown). They were stripped and reprobed with antibody to pIIIa to show that while all cell types were infected, HMGB1 was secreted only by corneal epithelial cells. We have added the scale to the revised S1C.*

SD-G: Text in these figures seems blurred.

*RESPONSE: Thank you, we believe the issue should be resolved with uploading of the high resolution images.*

**Reviewer #3:** The manuscript would benefit from thorough editing for grammatical correctness.

*RESPONSE: We have carefully reviewed the manuscript again for grammar. If there are additional errors that can be pointed out, we will be happy to correct them.*
